# A brief update on rabbit hemorrhagic disease virus.

**DOI:** 10.3201/eid0402.980236

**Published:** 1998

**Authors:** L Capucci, A Lavazza


### Reply to L.J. da Silva

**To the Editor:** Dr. da Silva's letter raises several important points. My article, however, was never intended to be comprehensive. The choice of which emerging infectious diseases to include was difficult, especially in a country where many endemic infections continue at a high prevalence and others, thought to be controlled, are reemerging.

As Dr. da Silva states, many reports (in Portuguese and English) discuss infectious diseases in Brazil; however, this information is rarely current. The information about measles in my article is a case in point. At the time of my article, an outbreak causing national concern was occurring in Brazil; it has since been controlled. A further problem is that the most detailed and reliable studies are generally of only a regional or local nature, for example, the recent excellent report by Merchan-Hamann (1) on the situation of endemic diseases in north and northeastern Brazil and other references cited by Dr. da Silva. To obtain current information at the national level and provide numerical data rather than merely discuss current trends, I focused on notifiable diseases.

As Dr. da Silva states, schistosomiasis has continued to decrease both in the number of cases and associated illness. Onchocerciasis has been restricted to a small focus in northern Brazil for many years, and a recent report of a new focus in the state of Goias has yet to be confirmed. In my opinion, neither infection could be considered emerging. An important helminthiasis that perhaps should be mentioned is Bancroftian filariasis with a main focus in Recife and minor foci in Belem and Alagoas. Because of traditional and novel control strategies, the number of cases is declining in all foci.

The information I used about hepatitis is confirmed by the National Reference Center on Viral Hepatitis of the Ministry of Health. Febrile illnesses in the Amazon are the great enigma and probably provide the cover for many new diseases that may still emerge. For example, only approximately 20% of blood slides taken from suspected malaria patients in the Amazon are confirmed as positive, which leaves at least one million cases of febrile illness per year undiagnosed. I am unaware of any data that show Mayaro and Oropouche viruses as the most common cause of these illnesses. Dr. da Silva's letter provides useful additional information on bacterial diseases, antimicrobial resistance, and a number of low-prevalence diseases that may in time prove to be important emerging infections.

**Hooman Momen**
Instituto Oswaldo Cruz, FIOCRUZ,
Rio de Janeiro, Brazil

### Reference:

1. Merchan-Hamann E. Diagnostico macroregional da situacao das endemias das regioes norte e nordeste. Informe Epidemiologico do SUS 1997;4:43-114.

### A Brief Update on Rabbit Hemorrhagic Disease Virus

**To the Editor:** We read with interest the paper by A. Smith et al. (Emerg Infect Dis 1998; 1:13-20) on calicivirus emergence from ocean reservoirs. Our attention was drawn particularly to the data and comments regarding rabbit hemorrhagic disease (RHD), a recently emerged and devastating disease of just one rabbit species, *Oryctolagus cuniculus*. We have been involved in RHD research and diagnosis since 1989. Like D. Gregg's laboratory at the Foreign Animal Diseases, U.S. Department of Agriculture, Greenport, USA, our laboratory at the Istituto Zooprofilatico Sperimentale della Lombardia e dell'Emilia, Brescia, Italy, was in 1991 designated a Reference Laboratory for RHD by the International Office of Epizootics (OIE), Paris, France. Although other aspects of the article by Dr. Smith and colleagues appear unclear (e.g., the fact that feline calicivirus is classified among human pathogens like Norwalk virus), we will confine our comments to a few main points concerning RHD virus (RHDV).

Is RHDV a calicivirus or a parvovirus? RHD is caused by a calicivirus (1-3). The articles cited by Dr. Smith date back to 1991 and are part of a book review promoted and edited by OIE (4). This landmark review includes papers from China and the United States supporting the parvovirus hypothesis and papers from Europe concluding that RHDV is a calicivirus. A retrospective reading of those articles may explain the reasons for the misinterpretation of some results. However, this occurred in 1991 and, after 7 years, more than 50 published articles consider RHDV a calicivirus. Actually, RHDV is one of the best characterized caliciviruses, and the publication of its full genome sequence in 1991 was the first of a *Caliciviridae* member (5).

Diagnostic tools have been developed by our and other laboratories (3,4,6). Thanks also to specific monoclonal antibodies produced towards RHDV and European brown hare syndrome virus (EBHSV) by our colleague E. Brocchi, we standardized different enzyme-linked immunosorbent assays (ELISAs) for the diagnosis of related diseases (4,6-8). In particular, we developed five different ELISAs for serology that allow the detection of antibodies specific for RHDV or EBHSV or that are cross-reactive. In addition, we can define the antibody response in rabbits and hares in terms of isotype-involved immunoglobulin M (IgM), IgA, and IgG (9). Today the main difficulty is the qualitative distinction between RHDV and rabbit calicivirus (RCV, a recently identified nonpathogenic calicivirus) antibodies because of the close antigenic profiles of these viruses (6). Finally, RHDV- and EBHSV-specific polymerase chain reaction has been developed in at least five laboratories besides ours. We have sent these reagents and/or diagnostic methods to at least 19 laboratories outside Italy, including Australia, New Zealand, and the United States.

Does RHDV infect humans? This question has arisen together with the prospect of using RHDV as a biologic control agent in countries like Australia and New Zealand, when they were free of RHDV. In Europe, where the disease naturally occurred and quickly spread, no particular control on human health was planned. In Italy only, between 1987 and 1990, hundreds of millions of rabbits died of RHD in regions where the average density of humans is very high. As a consequence of the use of the vaccine since 1991, the incidence of RHD among breeding rabbits decreased drastically and quickly. Nevertheless, the disease is still endemic, mainly in small farms and among wild rabbits. EBHS also is endemic in wild hares, and hunters are highly exposed to the virus since hares are their main target. However, neither in humans nor in animal species other than rabbits and hares have any diseases similar to RHD ever been reported. In relation to the likelihood of mild or inapparent infections, we used 100 human sera randomly selected from blood donors to carry out a preliminary standardization of an RHD-ELISA that has been periodically used to control the sera of the RHD laboratory staff. Very recently, we tested nine sera from laboratory personnel exposed to RHDV; again no positive result was noted by RHD-ELISA. These findings have limited epidemiologic value, but considering the high level of exposure of part of the sample, it is evident that RHDV infection in humans is unlikely to be the rule.

**Lorenzo Capucci and Antonio Lavazza**
Istituto Zooprofilattico Sperimentale della Lombardia e dell'Emilia, Brescia, Italy

### References

1. Murphy FA. Virus taxonomy. In: Fields BN, Knipe DM, Howley PM, editors. Virology. 3rd ed. New York: Lippincott-Raven Publishers; 1996. p. 15-57.
2. Ohlinger VF, Hass B, Meyers G, Weiland F, Thiel HJ. Identification and characterization of the virus causing rabbit hemorrhagic disease. J Virol 1990;64:3331-6.
3. Rodák L, Šmíd B, Valícek L, Veselý T, Stepánek J, Hampl J, et al. Enzyme-linked immunosorbent assay of antibodies to rabbit haemorrhagic disease virus and determination of its major structural proteins. J Gen Virol 1990;71:1075-80.
4. Capucci L, Scicluna MT, Lavazza A. Diagnosis of viral hemorrhagic disease of rabbits and European brown hare syndrome. Rev Sci Tech 1991;10:347-70.
5. Meyers G, Wirblich C, Thiel HJ. Rabbit hemorrhagic disease virus-molecular cloning and nucleotide sequencing of a calicivirus genome. Virol 1991;184:664-76.
6. Lavazza A, Capucci L. Viral haemorrhagic disease of rabbits. In: Office International des Epizooties, Paris, France, Manual of standards for diagnostic test and vaccine. Paris: the Office; 1996. p. 589-98.
7. Capucci L, Fusi P, Lavazza A, Pacciarini ML, Rossi C. Detection and preliminary characterization of a new rabbit calicivirus related to hemorrhagic disease virus but nonpathogenic. J Virol 1996;70;8614-23.
8. Capucci L, Frigoli G, Ronsholt L, Lavazza A, Brocchi E, Rossi C. Antigenicity of the rabbit hemorrhagic disease virus studied by its reactivity with monoclonal antibodies. Virus Res 1995;37:221-38.
9. Capucci L, Nardin A, Lavazza A. Seroconversion in an industrial unit of rabbits infected with a non-pathogenic rabbit haemorrhagic disease-like virus. Vet Rec 1997;140:647-50.

### Rabbit Hemorrhagic Disease

**To the Editor**: The recent article on calicivirus by Smith et al. (1) is misleading in its use of the study concerning human health aspects of rabbit hemorrhagic disease (RHD) by Mead et al. (2).

